# Interactive Rooting Towers and Behavioural Observations as Strategies to Reduce Tail Biting on Conventional Pig Fattening Farms

**DOI:** 10.3390/ani11113025

**Published:** 2021-10-21

**Authors:** Anne Kalies, Johannes Baumgartner, Martin Beyerbach, Milos Stanojlovic, Tobias Scholz, Franziska Richter, Alexandra von Altrock, Isabel Hennig-Pauka

**Affiliations:** 1 Clinic for Swine, Small Ruminants, Forensic Medicine and Ambulatory Service, University of Veterinary Medicine Hannover, Foundation, Bischofsholer Damm 15, 30173 Hannover, Germany; anne_kalies@yahoo.de; 2 Institute of Animal Welfare Science, Department for Farm Animals and Veterinary Public Health, University of Veterinary Medicine Vienna, Veterinärplatz 1, 1210 Vienna, Austria; johannes.baumgartner@vetmeduni.ac.at; 3 Institute for Biometry, Epidemiology and Information Processing, University of Veterinary Medicine Hannover, Foundation, Bünteweg 2, 30559 Hannover, Germany; martin.beyerbach@tiho-hannover.de; 4 Department of Pharmacology, Toxicology and Pharmacy, University of Veterinary Medicine Hannover, Foundation, Buenteweg 17, 30559 Hannover, Germany; milos.stanojlovic@tiho-hannover.de (M.S.); franziska.richter@tiho-hannover.de (F.R.); 5 Chamber of Agriculture of North Rhine-Westphalia, Haus Duesse 2, 59505 Bad Sassendorf, Germany; tobias.scholz@lwk.nrw.de; 6 Field Station for Epidemiology, University of Veterinary Medicine, Hannover, Foundation, Buescheler Straße 9, 49456 Bakum, Germany

**Keywords:** welfare, environmental enrichment, tail manipulation, swine, biter

## Abstract

**Simple Summary:**

In Europe, tail docking in swine is prohibited as a routine measure, but the risk of tail biting outbreaks is increased in pigs with intact tails. An important measure to minimise this risk is the provision of enrichment material, which is challenging in conventional confinement buildings with fully slatted floors. In this study, the effect of an interactive straw-filled rooting tower was tested under field conditions with respect to the prevalence of tail lesions and behaviour. Although tail biting could not be prevented in pigs with intact tails, tail lesions were less frequent and less severe compared to pigs which had no access to straw but were only exposed to the stationary tower as a placeholder (control group). Increased manipulation of the rooting tower around feeding times reflected the typical porcine ambition for simultaneous feed uptake in a group. In addition, tail manipulation was less common than head manipulation in pigs with access to the interactive tower. The rooting tower can be used in addition to other measures as a preventive and intervening tool to deal with tail biting in pigs with intact tails, and on farms with fully slatted floors.

**Abstract:**

Eight pens (25 pigs/pen; *n* = 200) provided with an interactive straw-filled rooting tower (experimental group) and five pens (25 pigs/pen; *n* = 125) with a stationary (fixed) tower without straw (control group) were compared within three fattening periods on a conventional farm with fully slatted flooring. The effectiveness of the tower to trigger favourable behaviour in feeding and outside feeding periods was assessed. The incidence of deep tail injuries was lower in the experimental group (experimental group: Odds Ratio 0.3, *p* < 0.001) and was influenced by the batch (Odds Ratio: 2.38, *p* < 0.001) but not by pen and sex. In spring, most pens were excluded due to severe tail biting. Tail injury scores were more severe in the control group in weeks 5, 6 and 7 compared to the experimental group (*p* = 0.002, *p* < 0.001, *p* < 0.001, respectively). Tower manipulation was more frequent during feeding compared to outside feeding time (*p* = 0.002). More head than tail manipulation occurred in the experimental group (*p* = 0.03). The interactive tower as the only measure was not appropriate to reduce tail biting sufficiently in pigs with intact tails on a conventional fattening farm. Of high priority to prevent tail biting outbreaks was the early detection of biting pigs.

## 1. Introduction

Tail biting is a highly challenging welfare issue in pig husbandry, causing massive economic losses to the pig industry [1]. While tail docking does not completely prevent tail biting, it is routinely carried out to reduce its risk. In fact, 90–95% of pigs within the European Union are still tail-docked although Council Directive 2008/120/EC demands that tail docking must not be carried out routinely and that other measures shall be taken to prevent tail biting. Importantly, the Directive specifically demands the elimination of environmental and management risk factors [2,3]. In Sweden and Norway, the ban on tail docking has been complied with for several years. Reports regarding the prevalence of tail lesions in slaughter pigs vary, depending on the scoring method, between 1–7% [4,5,6].

Taylor et al. (2010) described three different types of tail biting: the most often reported type is the explorative two-stage tail biting. During the first ‘pre-injury stage’, pigs displayed their natural rooting, chewing and gnawing behaviour, which can be directed towards pen mates in a barren environment. This initial phase is followed by the ‘injury stage’, where pigs are additionally keen to perform tail biting due to a visual stimulus and the taste of blood. Injuries varied between only small bite marks up to the complete loss of the tail. The second type of tail biting may occur as a consequence of a deprivation of specific resources, e.g., water, feed or space. It was characterised as a sudden and forceful biting behaviour [7]. The third type of tail biting was described as an aggressive behaviour of single individuals behaving in an abnormal way. In that case, no etiological cause for biting was identified. Hence, this type of biting was defined as a behavioural disturbance of unknown origin [7]. A fourth type of tail biting has been recently suggested by Valros et al. (2017), which is characterised by its sudden onset and epidemic-type clustering in specific pens. Sudden changes in the environment or in the perception area of the pigs are considered to be the cause of biting outbreaks [8]. Regardless of the different types of tail biting, tail docking most efficiently reduces the probability of being bitten [9]. This invasive procedure neither addresses the underlying causes nor does it comply with Council Directive 2008/120/EC. Thus, more research on alternative strategies is essential. The three most common interventions in pigs with intact tails used by Swedish farmers in cases of tail biting outbreaks were removal of biters, separation of injured pigs and additional provision of straw [6,10].

Tail biting is regarded as a multifactorial problem [11]. Several on-farm risk factors have been described, but initiating trigger factors for outbreak situations can often not be elucidated in complex field conditions. Insufficient environmental enrichment has a major impact on tail biting incidence, together with indoor and outdoor climate, the general health status of the animals, stocking density, herd size, feeding system and water supply [7,12]. Software-based advisory tools were developed in several EU countries to detect risk factors on farms and to produce herd-specific solutions in order to reduce the incidence of tail biting in the pig industry [13,14,15,16,17]. The German tool SchwIP was used in this study on the farm prior to the experiment. SchwIP is based on a questionnaire to be answered by the farmers addressing known risk factors, an inspection of the pigs in their farm environment and a farm-individual feedback and advice how the incidence of tail biting could be reduced [17,18]. An evaluation of SchwIP records from 25 farms resulted in stocking density, suckling piglet losses, numbers of litters mixed after weaning and the daily weight gain as major risk factors for tail lesions in weaner pigs [19]. The proportion of pigs with tail lesions at slaughter could be reduced over time on farms receiving advice, but were on average still higher than 25% [20]. By using SchwIP, the calculated risk for tail biting outbreaks was reduced on respective farms but was insufficient to prevent tail biting. So far, the tool does not address the complex relationship between different factors in a given environment [18].

One requirement to guarantee minimum welfare standards for pigs is the permanent access to a sufficient quantity of material to enable explorative behaviour (Commission Recommendation (EU) 2016/336, Council Directive 2008/120/EC), which is limited in conventional housing systems due to economic, technical and hygienic reasons. On conventional farms, at least the legal requirements for housing conditions are implemented. Appropriate enrichment material should be ingestible, deformable, destructible, chewable and odorous, because its property has a high impact on the damaging behaviour of pigs [21,22]. However, enrichment materials cause potential difficulties in slatted systems with liquid manure handling facilities [23], so that objects (e.g., wooden pendular beams, cross of chains, lifting beams, ropes, suspended plastic toys) were often chosen as alternatives [24]. Nevertheless, the attractiveness of these objects in general is limited [25,26,27]. Comparison between farms using chains, plastic or wood revealed more damaging behaviour and more tail lesions on farms using chains as enrichment objects [22]. Wooden posts are frequently used on pig farms, but the type of wood influences the attractiveness for pigs and there is lack of evidence that wooden posts can reduce tail or ear biting [28].

In addition to the properties of enrichment material or objects, the way they are offered, e.g., location, amount and frequency, also has an impact on the final effect of reducing tail biting [29]. Restricted access to point-source enrichment material, for example, can trigger aggressive behaviour due to competition [30]. Free toys presented loose on the floors are soiled and quickly become unattractive, while hanging toys seem to be more attractive, and rotating different objects seems to additionally increase the attractiveness of the tool [31]. To guarantee that pigs are continuously interested in the enrichment material, the objects and material should be exchanged before pigs lose interest [32]. This is especially important in objects which are not deformable or destructible, such as plastic balls and tubes [27,29]. Insufficient renewal of enrichment objects and material is one of the most commonly found risk factors for tail biting [13,14,27,33,34]. A high enrichment replenishment rate (daily or ad libitum) was shown to improve growth rate and reduce damaging behaviour compared to a thrice-weekly replenishment rate [35]. On conventional farms, enrichment material can be provided by racks on a routine basis, minimising the risk of blockage of the manure system [36]. The consumption of straw from racks must initially be learned by pigs and can be hampered when the rack is too high for young pigs with low withers height [36]. This practice of straw provision is promoted by the requirements for enrichment material fixed in the German national action plan for banning tail docking. Out of the three largest pork-producing regions, USA, China and the EU, only the EU has legal requirements to provide enrichment. Effective solutions to implement enrichment on farms are driven by improvements in productivity, so that innovative approaches are urgently needed [29].

The hypothesis of the study was that an interactive object, which can be operated by the pigs, followed by release of enrichment material as a reward maintains its attractiveness better than an unchanging object. The interaction of fattening pigs with intact tails with a straw-filled manoeuvrable rooting tower (termed interactive tower) was compared to a fixed tower without straw (termed stationary tower) on a commercial farm. It was evaluated (i) how long the towers stay attractive for the pigs, (ii) if tail biting outbreaks can be prevented or delayed or whether the number of affected individuals was reduced, so that intervention is still possible for the farmer, (iii) if exploratory behaviour directed to the tower is increased and pen mate-directed behaviour decreased. It was expected that the interactive tower (experimental group), which combines the favourable properties of being movable and of providing edible material was more and longer attractive for pigs compared to the stationary tower (control group). In addition, the influence of the tower during feeding time on interaction between pen mates was evaluated. It was hypothesised that pigs, which were not able to eat at the beginning of feeding times due to the restricted number of feeding places would manipulate the tower and show less competitive pen mate interactions. The interactive tower has been previously tested in tail docked pigs in its full function and without straw, but the low prevalence of tail biting in the examined groups allowed no assessment of its effect [37]. In this former study, climate factors such as high ammonia concentrations and air draught were identified as trigger factors for tail biting [37]. In our study presented here, we assessed the pigs’ interest in the interactive or stationary towers during the fattening period, the influence on pen mate-directed behaviour and the incidence of tail lesions. According to the four criteria of successfully employed enrichment material [38], our study focused on the aspects whether the interactive tower supports species-specific behaviour and whether tail injuries as one important health issue in fattening pigs can be reduced.

## 2. Materials and Methods

### 2.1. Study Location and General Management of Tail Biting Risk Factors

The study protocol was reviewed by the Animal Welfare Officer and corresponding legal entities before the start of the study (TVO-2014-V-1). Thereby, it was determined that the study did not require permission under the German legislation on animal testing. The study was performed within one year in one compartment of a commercial fattening farm in Brandenburg, Germany. In total, the farm had 1890 fattening places in eleven compartments with six pens each and 25 pigs per pen.

**Figure 1:**
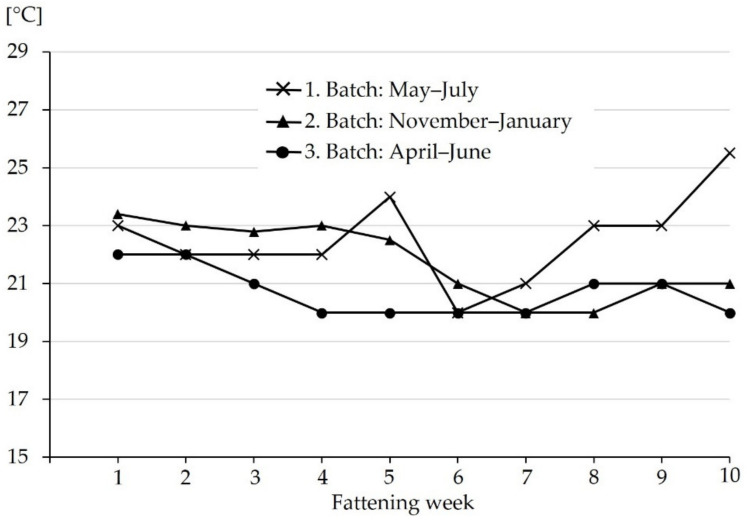
Indoor temperature in the different fattening weeks in the study compartment. The three different fattening periods (batches) in different seasons are shown.

Prior to starting the study, two pens in two compartments with pigs of a 70 kg body weight were checked by ‘SchwIP’, a software based tail biting intervention tool established by the Institute of Animal Welfare and Animal Husbandry of the Friedrich-Loeffler-Institute, Celle, Germany [18]. Seven risk factors for the occurrence of tail biting were identified: ear biting in 0.8% of examined pigs, mild clinical signs of respiratory disease, varying flow rates of water at the nipple drinkers, no enrichment material, feeding four times a day, environmental temperature 25–26 °C, no wallowing space. The first three risk factors were eliminated before the start of the study as follows: (i) only batches of pigs without ear and tail injuries were included in the study, so that ear biting as a risk factor itself was excluded from the outset of the study, (ii) respiratory health was improved before the start of the study by immediate treatment of individual coughing pigs in the nursery of the upstream farrowing farm, (iii) nipple drinkers were exchanged to guarantee optimal flow rates in all pens. The risk factors “feeding system” and “lack of wallowing space” could not be eliminated in this system. The risk factor “lack of enrichment material” was the focus of this study. Indoor target temperature as an important risk factor was adjusted as close as possible to the recommendations for the respective age-groups (18–26 °C) [39]. As shown in Figure 1, temperatures in heavy fatteners were above the recommendations in the summer batch, so that high temperature as a risk factor could not be eliminated during the entire fattening period in this batch.

### 2.2. Animals and Husbandry

The cross-bred pigs originated from a farrowing farm with Danish Landrace x Danish Yorkshire sows and Pietrains used as paternal line. Sows farrowed in farrowing crates. For each trial period, piglets from one farrowing batch were reared in one large group of 85 pigs after a four-week lactation period until being moved to the fattening farm. On arrival at the fattening farm, pigs were age 77 to 87 days (approximately 12 weeks of age). Pigs had been vaccinated once against *Mycoplasma hyopneumoniae* and Porcine Circovirus type 2 at the age of three weeks. Within one year, a total of 155 female and 170 castrated male pigs with intact tails (*n* = 325) were examined in three fattening periods (trial periods: May–July, November–January, April–June). Pigs were assigned randomly to two different environmental enrichment intervention groups: pigs housed in pens equipped with a manoeuvrable rooting tower regularly filled with straw (interactive tower, total of *n* = 200 pigs distributed over eight mixed-sex pens, 48% female and 52% male, experimental group) and pigs housed in pens containing a fixed rooting tower without straw as the control group (stationary tower, *n* = 125 pigs distributed over five mixed-sex pens, 47% female and 53% male). Information about animals in the individual pens and batches is given in Table 1.

On arrival, the pigs were weighed and ear-tagged for individual identification. The average body weight of the pigs at arrival was 34.6 ± 6.3 kg. Pigs were allocated to the pens, aiming at a balance between the number of male and female pigs within one pen. The health status and the condition of the tail of each pig was recorded. During the study, the health status was checked twice a day and tails were scored on a weekly basis every Monday. Pigs were weighed a second time between fattening weeks 7 and 10 at fattening day 51 (first batch), day 52 (second batch) and day 74 (third batch). The study compartment contained six pens with fully slatted concrete floors, three on each side of a central corridor with solid partition walls between the pens. Twenty-five pigs were allocated to each pen providing 0.98 m^2^ space per pig. Pens with the interactive (experimental group) and with the stationary tower (control group) were in the same study compartment.

**Figure 2:**
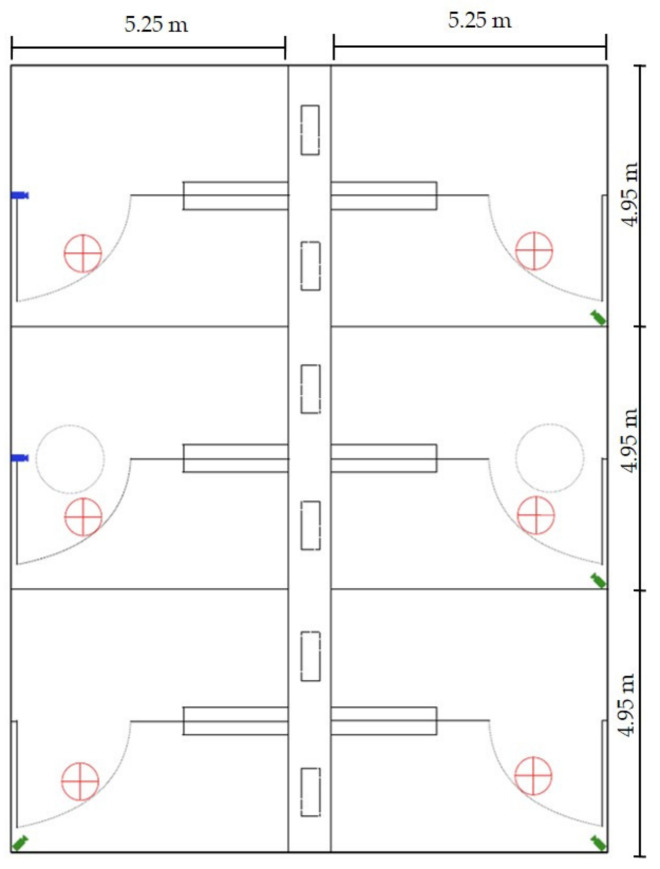
Compartment on a commercial fattening farm with identical fully slatted pens on each side of a central corridor. Pens are divisible by a turnable wall (radius depicted by dotted line). Cameras (Sony^®^ 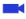, EuroTech^®^ 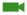) were fixed to the ceiling. 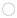 exhaust air fan, 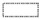 inlet air flap, 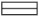 sensor-equipped feeding trough, 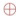 rooting tower).

The fresh air supply was controlled throughout six adjustable valves in the ceiling above the central corridor and removed via two ventilators located above the animal area (Figure 2). Dung was removed from pens automatically via the slatted floors and reciprocal outflow slurry. Liquid manure was collected via a pipe system 0.2 m in diameter in a pre-slurry pit of 25 m^3^ reservoir capacity before it was pumped at regular intervals to the final storage facility for liquid manure with 2400 m^3^ reservoir capacity. Ammonia concentration measured in the first and sixth week of the fattening period was <5 ppm. The mean light intensity was 85 lux. Light was switched on for 13.5 h during the day (07:00–20:30). Room temperature and relative air humidity were measured weekly and ranged from 20.0–25.5 °C (Figure 1) and from 61–75%, respectively.

Pigs had free access to water provided by four nipple drinkers per pen and were fed automatically by a liquid feeding system (WEDA Dammann and Westerkamp GmbH, Lutten, Germany) four times a day (07:30, 12:00, 17:00, 20:00) with multiple feed rations provided at intervals of approximately two minutes per feeding time until satiation. One sensor-equipped feeding trough with a length of 200 cm and accessible from two sides was located in the middle of each pen. For approximately two pigs, one feeding place (33 cm) existed in this system (ratio of 2.1 pigs per feeding place). Feed composition was adjusted according to the weight of the pigs into three periods (up until 60 kg body weight, 60–80 kg body weight, and up until slaughter). Diet mainly consisted of corn cob silage and cereal flour. At the beginning of the fattening period, a diet with 13.3 MJ/kg, 16.8% total protein and 3.8% crude fibre was fed for seven weeks. In the following three weeks, this feed was mixed with the finishing diet (12.7 MJ/kg, 16.1% total protein, 3.8% crude fibre) in a ratio of 3:1 (first week), 1:1 (second week), 1:3 (third week). Subsequently, the finishing diet was fed until the end of the fattening period. A movable chain with two plastic bars at its end anchored at the upper part of the side wall as well as a movable plastic ball anchored at the floor were routinely provided in every pen as enrichment objects.

### 2.3. Set-Up of Interactive Rooting Tower (“Duesser Wuehlturm”)

**Figure 3:**
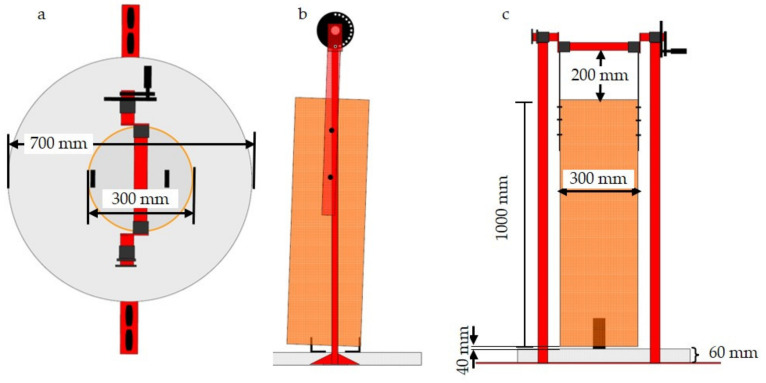
Dimensions (in mm) and layout of the rooting tower “Duesser Wuehlturm”. (**a**) tower viewed from above, in which the pipe is not centred in relation to the concrete base plate. The diameters of the concrete base plate and the pipe, which can be straw-filled, are depicted, (**b**) lateral perspective with the pipe not being centred. The swing radius of the pipe is restricted by rectangular brackets fixed on the base plate, (**c**) frontal perspective showing the heights of the base plate, the pipe and the distance between the pipe and steel frame.

**Figure 4:**
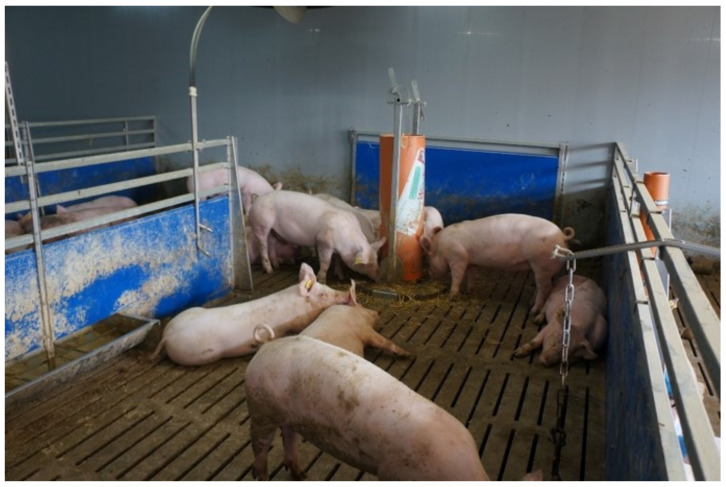
Pigs in a pen equipped with the interactive tower.

The interactive rooting tower, called “Duesser Wuehlturm” was designed by the Chamber of Agriculture in North Rhine-Westphalia, Haus Duesse, Germany. A plastic pipe measuring 100 cm in length with a diameter of 30 cm was suspended in a stainless steel frame and fixed to a 0.38 m^2^ round concrete basis plate. The two-directional swing radius of the pipe was restricted by two rectangular brackets left and right to the centre of the ground plate. The suspension at the top allowed a height-adjustable gap between the platform and the plastic tube, which could be varied by a stainless steel disc with 18 holes fixed to the steel frame. The axis could be circled by using a crank lever, which could be fixed at the respective height by a metal pin in one of the 18 holes to the disc. As a consequence, the plastic pipe could be lowered or lifted so that the gap between the plastic pipe and concrete plate could be varied between 10 and 80 mm (Figure 3). The suspension of the plastic pipe at the top remained flexible in a tube sleeve so that pigs could push against the pipe and move it forward until it was stopped by the bracket at the respective side. By this movement, the straw inside the pipe slipped slowly downwards until reaching the gap. During the study, the gap was adjusted to a height of 40 mm for release of an appropriate amount of straw over time, which could be taken up by the pigs and did not clot the slats as published previously [37]. When pigs moved the straw-filled pipe when rooting, straw was released to the floor’s plate where it could be explored, manipulated and eaten (Figure 4). It is recommended by the inventor to fill the rooting tower manually at least once a day with 30–50 g of fresh straw per pig. Before the start of the study, a straw length of 50 mm was found to be appropriate to be removed from the tower by pig manipulation without causing clogging inside the tower or clogging of the slurry pumps. This was in accordance with another published study [37]. The tower was controlled twice a day and re-filled with straw before running empty. A daily straw amount of 50 g per pig, i.e., 1250 g per pen was estimated. This amount corresponded to approximately 41 L of loose straw per pen. The tower pipe was completely filled with approximately 70 L straw, so that twice a day the pipe could be filled with much more than the recommended amount. The tower was, therefore, never empty during the study. The stationary tower served as a control. It was not filled with material, was not manoeuvrable and served as a placeholder to guarantee similar activity space in both groups.

### 2.4. Scoring, Behavioural Analysis and Interventions

#### 2.4.1. Examination of Tail Lesions

All pigs were individually examined for tails lesions on every Monday of each week. Tail lesions and losses were assessed by clinical adspection and scored (Table 2) for (i) tail lesions (score 0–3), (ii) tail losses, defined as any reduction in physiological tail length (score 0–3) and (iii) acute tail injuries with fresh blood visible (score 0 or 1). Assessment of tail injuries was always performed by the same observer and followed a modified German scoring system.

#### 2.4.2. Behavioural Observations

**Figure 5:**
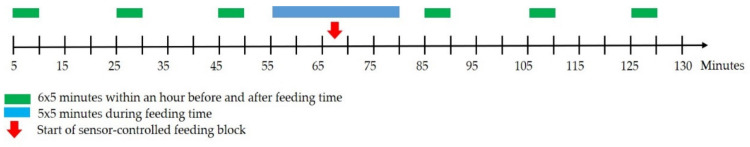
Schematic timeline of video-based observation periods (in minutes) around feeding time, which were performed twice a day during the noon and afternoon feeding. Five-minute intervals outside feeding are indicated in green and during feeding in blue.

The behaviour of the pigs was analysed retrospectively for each pen from video recordings every second week (weeks 2, 4, 6, 8 and 10 of the fattening period) in accordance with an ethogram modified from Zonderland et al. [42] as shown in Table 3. Digital video cameras (Sony SUPER HAD CCD II 700, Tokyo, Japan and Lupustec EuroTECH LULE138 LA 4, LUPUS—Electronics^®^ GmbH, Landau, Germany) were fixed to the ceiling. Data reception and storage were performed by a digital recorder (EuroTECH LULE800D1, LUPUS—Electronics^®^ GmbH). The behaviour of the pigs in the experimental and control pens were videorecorded from 07:00 to 20:00 always on the same day (Wednesday) every second week. Video recordings from Wednesday in fattening weeks 2, 4, 6, 8 and 10 were analysed retrospectively covering 110 min per day. Continuous sampling of defined behavioural parameters displayed in the group was performed from video sequences within five-minute periods. Prior to the beginning of the evaluation of each five-minute period, all pigs within the field of vision were counted. Predefined behaviour in the pig group was counted within the following five minutes at group level and not assigned to individual pigs. In total, 22 five-minute periods per observation day in every pen were evaluated, which resulted in a total of 110 min observation time per observation day. This time period was distributed in five subsequent five-minute periods in the morning (25 min) and five subsequent five-minute periods (25 min) in the evening during feeding time. In addition, within one hour before and one hour after the respective feeding times, three five-minute periods (2 × 15 min in the morning and in the evening; in total 60 min) interrupted by 20 min each were analysed, respectively (Figure 5). Time periods around feeding at noon between 11:00 and 13:00, and in the afternoon between 16:00 and 18:00 were evaluated. Behavioural patterns for statistical evaluation were fighting, biting and manipulation of towers and pen mates as defined in Table 3. Activities which were interrupted for a time span of at least five seconds were assessed as a new action. Finally, behavioural data were transformed to an average frequency of a specific behaviour per pig in one pen within five minutes (number of actions in a pen divided by the number of pigs). In case a tail biting outbreak occurred in one pen, additional interventions were taken, which are described in Section 2.4.3, and the pen was removed from all data analysis until the end of the fattening period.

#### 2.4.3. Interventions at Occurrence of Severe Tail Biting

If severe tail biting occurred in a pen, specific interventions were applied. Severe tail biting was defined as an outbreak, when at least nine pigs (more than one third of pigs in the pen) suffered from acute bloody tail lesions at the same observation time. In total, *n* = 9 and *n* = 7 pigs in pens with the interactive and stationary tower, respectively were identified as tail biters and separated (Table 1). If severe tail biting occurred, the tower was changed to an interactive tower (manoeuvrable and filled with straw) in the control group. Victims were separated within the pen by partitioning off the pen into equal sections with a partition wall regardless of the group. Injured pigs were immediately treated by a veterinarian. Pens with tail biting outbreaks were excluded from further evaluation.

### 2.5. Statistical Analyses

All data were visualised and statistically evaluated by the SAS^®^-System (Version 9.3, SAS Institute Inc., Cary, NC, USA) and SigmaPlot (Version 14.0., Systat Software, San Jose, CA, USA).

The statistical unit of behavioural data was the pen. The five-minute periods during feeding were averaged and compared to those five-minute periods before and after feeding, which were combined and also averaged. The distribution of the respective five-minute periods is depicted in Figure 5. All behavioural data sets were tested for normal distribution (Shapiro–Wilk test) prior to the choice of statistical test.

Comparison of behavioural data between experimental groups at feeding and in time-periods outside feeding (before and after feeding) were performed with the Mann–Whitney U test. Wilcoxon’s signed rank test was used to compare behavioural data between time points at feeding or outside feeding periods within the experimental groups. In addition, selected behaviours such as head and tail manipulation were compared within the groups using the Wilcoxon’s signed rank test.

In addition, the ratio of tower manipulation to total manipulating behaviour was calculated for each observation day and group during feeding and outside the feeding period. Data were normally distributed (Shapiro–Wilk Test *p* > 0.05). For fattening weeks 2 and 4, the number of observations was sufficient for a two-way repeated measures analysis of variance (ANOVA) with factors group (experimental versus control) and feeding period (feeding versus outside feeding). Pairwise multiple comparison procedures (Holm–Sidak method) were performed to identify significant differences.

The association between the prevalence of tail injuries and the group (experimental versus control) was evaluated in a multiple logistic regression model. The dependent variable was the dichotomised parameter tail injury with one category including pigs with intact tails or only superficial skin scratches (score ≤ 1) and the other category with a score > 1 (Table 2). Independent variables were: (i) the group, (ii) time period reflecting the seasons summer, winter and spring (1–3), (iii) pen (1–6) and (iv) sex. The variance inflation factor was determined to test variables for multicollinearity.

The ordinal scores for tail injury and tail loss were tested in different fattening weeks within each experimental group using the Kruskal–Wallis test, followed by Dunn’s Method post hoc. Comparison of scores for tail injuries and tail loss between experimental groups at specific time points was made with the Mann–Whitney U test.

The number of pens excluded due to severe tail biting outbreaks as well as proportions of pigs with tail injuries were compared between experimental and control group using the Chi-square test.

The association between the average daily weight gain and the group (experimental versus control) was evaluated in a multiple linear regression analysis. Independent variables were: (i) the group, (ii) time period reflecting the seasons summer, winter and spring (1–3), (iii) pen (1–6) and (iv) dichotomised variable tail injury (score ≤ 1, score > 1).

For all statistical evaluations, *p* values below 0.05 were reported as statistically significant.

## 3. Results

Mean average daily weight gain was 1008 ± 153 g in the control and 1057 ± 126 g in the experimental group. Multiple linear regression resulted in a significant influence of the experimental group on the average daily weight gain of individuals (*p* = 0.01, interactive tower associated with higher weight gain), while the pen, batch and tail injury had no influence. The variance inflation factor was close to 1 for all tested variables, indicating that there was no multicollinearity of the variables. Tail injuries and losses were recorded in all pens. Identified biting pigs were removed from the pens and bitten pigs were treated. In total, three animals died (<1%). Information about animals in the pens of the experimental and control group are recorded in Table 1.

### 3.1. Tail Injuries Were Reduced in Pens with the Interactive Tower

After arrival of the piglets to the fattening farm in the first batch, the first scoring resulted in 31 piglets with reduced tail length (no intact tail) but without injuries. These piglets were excluded from statistical evaluation of tail injuries during the fattening period. Tail injuries (score > 1, Table 2) were detected in 60.5% pigs of the experimental and 80.3% of the control group (Chi-square test *p* = 0.0003). In a multiple logistic regression model, the prevalence of tail injuries (score > 1, Table 2) was associated with the group (experimental and control). Pigs in the experimental group had on average a 70% lower risk of experiencing tail injuries than pigs in the control group (Table 4). The later batch (i.e., towards the spring season) increased the average risk of tail injuries by 2.4 times (*p* = 0.001), while sex and pen had no effect in this model. The variance inflation factor was close to 1 for all tested variables, indicating that there was no multicollinearity of the variables. Batch and group independently affected the incidence of tail injuries.

**Figure 6:**
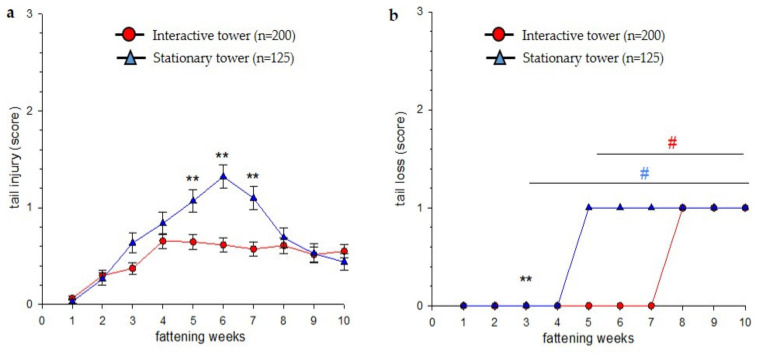
Scoring of tail injury and tail loss in the experimental and control group (number of pigs (*n*) indicated in the legend). (**a**) Tail injuries (score 0–3) of individual pigs in the different groups are presented as means ± SEM. In case more than nine pigs in a pen suffered from acute tail injuries, the respective pen was excluded from evaluation. (**b**) tail loss presented as median (score 0–3) of individual pigs. Statistics were performed using a non-parametric test. Significant differences are indicated: ** *p* < 0.01, Mann–Whitney U test, experimental versus control group. # *p <* 0.05, Dunn’s method indicated for all weeks significantly compared to week 1 (stationary tower in blue, interactive tower in red).

Pigs in the experimental group showed lower tail injury scores in weeks 5, 6 and 7 (Mann–Whitney U test, *p* = 0.002, *p* < 0.001, *p* < 0.001, respectively) compared to pigs in the control group (Figure 6a). Scores for tail losses became significant in week 3 for pigs in the control group and in week 5 for pigs in the experimental group compared to the first scoring in the first fattening week (Kruskal–Wallis effect throughout the fattening period within the group, *p* < 0.001 independent of type of tower; Dunn’s method, *p* < 0.05 for respective weeks, Figure 6b). At week 3, the loss scores were lower in the experimental group than in the control group (Mann–Whitney U test, *p* = 0.002). Two out of eight pens in the experimental group and four out of five pens in the control group were affected by severe tail biting outbreaks. This difference between the groups was significant (Chi-square test, *p* = 0.05). At the peak of outbreaks, biting pigs could be identified and removed as part of the intervention (1–3 biting pigs per outbreak).

### 3.2. The Interactive Tower Was More Attractive Especially during Feeding, and the Animals Favoured Head over Tail Manipulation

**Figure 7:**
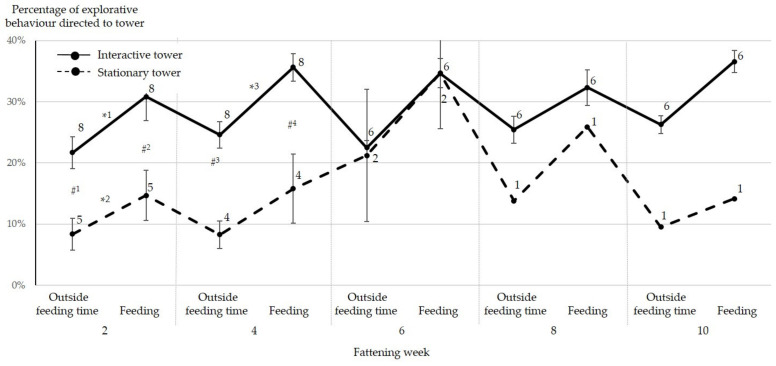
Percentage of time spent for manipulation of the tower in the total manipulation time in the control and experimental group. Data of pens in the different groups are presented as means ± SEM in different weeks of fattening outside feeding times and during feeding. Numbers of evaluated pens are depicted next to the marks. Several pens were removed from data analysis due to severe tail biting outbreaks which required interventions such as providing additional straw and separating the pen. * significant difference between time points within group, # significant difference between groups at respective time point. *^1^ *p* < 0.001, *^2^ *p* = 0.017, *^3^ *p* = 0.001, #^1^ *p* = 0.029, #^2^ *p* = 0.011, #^3^ *p* = 0.004, #^4^ *p* < 0.001 (Pairwise multiple comparison procedure, Holm–Sidak method).

**Figure 8:**
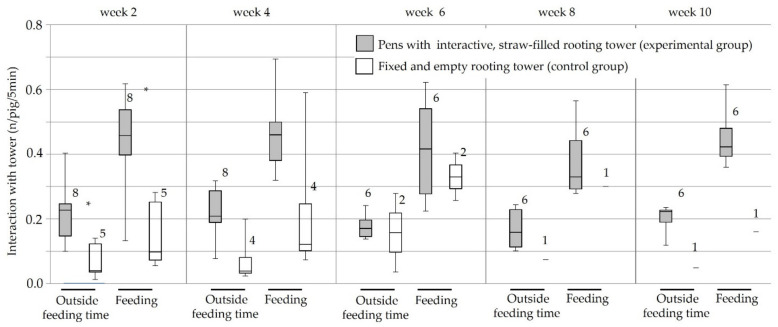
Average frequencies of tower manipulations per pig within five minutes in the experimental (interactive tower) and control (stationary tower) group. Boxes represent the 50% between 25% and 75% quartiles. The line inside the box indicates the median. The top and bottom lines denote maximum and minimum values. Observation time (Outside feeding time = before and after feeding and during feeding) and week of fattening is indicated. Numbers of evaluated pens are depicted next to the bars. Several pens were removed from data analysis due to severe tail biting outbreaks which required interventions such as providing additional straw and separating the pen * *p* < 0.05, Mann–Whitney U test; experimental versus control group.

In order to reveal mechanisms showing how the interactive tower reduced the frequency and severity of tail injuries, behavioural parameters were analysed up to the time point when severe tail biting required interventions for the respective pen (as indicated in Table 1). A descriptive summary of behavioural data is given in Table 5. In week 2, the fraction of the total manipulation time in which pigs interacted with the tower (% of explorative behaviour directed towards the tower, Figure 7) was higher in the experimental group compared to the control group, and higher during feeding time compared to outside feeding time. For the experimental group, this continued in week 4, but for the control group, manipulation was no longer different during feeding compared to outside feeding (2 × 2 repeated measures ANOVA, followed by Holm–Sidak method, Figure 7). Single pens in the control group experienced severe tail biting in fattening weeks 2, 4, 5 and 6. The respective pens were excluded from data analysis due to interventions precluding further meaningful statistical comparison between the tower types. Confirming this observation, in the experimental group, absolute manipulation of the tower was more frequent during feeding than outside feeding during the entire fattening period (Wilcoxon-signed rank test, weeks 2, 4: *p* = 0.008, weeks 6, 8, 10: *p* = 0.03, Figure 8). In week 2, manipulation of the interactive tower was more frequent than that of the stationary tower (Mann–Whitney U test, outside feeding time: *p* = 0.02; during feeding: *p* = 0.01, Figure 8). Fighting behaviour or biting was only rarely observed and did not differ between pens in the experimental and the control group (see descriptive summary of observation period in Table 5). The evaluation of behaviour within each group revealed that in pigs in the experimental group, head manipulation occurred significantly more often than tail manipulation, irrespective of feeding or outside feeding time (Wilcoxon-signed rank test, weeks 2, 4: *p* = 0.008, weeks 6, 8, 10: *p* = 0.03), while in the control group, there were no differences in the frequency of exploration of different body regions.

## 4. Discussion

In the present study, the practicability of an interactive tower as a preventive measure to reduce the risk of tail biting outbreaks was assessed in a commercial fattening herd with pigs with intact tails. The interactive “Duesser Wuehlturm” combined the recommended positive effects of being a manoeuvrable object and the provision of straw as enrichment material [21]. Previous studies regarding strategies to reduce tail biting focused on weaner pigs with intact tails [43,44,45,46,47,48], while studies in fattening pigs with intact tails are rare. Tail biting outbreaks are considered to be inevitable also on well managed farms due to various risk factors which can vary between different time periods and in their constellations [6,49]. This is the reason for the varying success rate in rearing pigs with intact tails between different batches on the same farm. Additionally, in our study, a significant batch effect on the incidence of tail injuries was found. Two out of eight pens in the experimental group were finally affected by severe tail biting outbreaks although the farmer had tried to avoid all tail biting causes found by the specific risk assessment report of SchwIP prior to the start of the study. This reveals the need for further adaptation of housing conditions of fattening pigs with intact tails (i.e., stocking density, feeding places, air quality). Prior to the start of the study, the feeding system was considered as a risk factor but could not be changed. Sensor-controlled feeding systems bear the risk of demixing liquid feed with the consequence of a too-low dry matter content. Feed hygiene can be a problem when feed remains in the trough and uncontrolled fermentation takes place [50]. Feeding valves and the consistency of feed were regularly controlled throughout the study, so that the occurrence of this risk factor might be of low probability. Other authors evaluated the ratio of pigs to feeding place in this system and tested ratios wider than 4:1 [51,52]. In our study, approximately two pigs shared one feeding space, so that this risk factor might also be of lower importance. The space of 0.98 m^2^/pig can be considered as high and is comparable to the Swedish housing conditions providing 0.9 m^2^/pig [6]. Of higher importance might, therefore, be pen structuring and climate control in this system. The farm had no cooling devices for pigs and was, therefore, not adapted to hot weather. Thermoregulation in pigs is hampered in confined livestock buildings due to the limitation of evaporative cooling [53]. Fattening pigs and sows suffer most from heat stress in hot weather. In our study, four out of six pens, which had to be excluded from further evaluation due to a tail biting outbreaks were in the batch in spring (Table 1). In the respective year, this time period was characterised by large variations in temperature and the amount of precipitation. The regulation of forced ventilation to adjust indoor climate is most challenging in periods with sudden outer climate temperature changes. An additional risk factor, which was not addressed by SchwIP, might be the light intensity on this farm. Some empirical observations of farmers indicate that the obligatory 80 lux in Germany could be a risk factor in some cases. The EU Directive 2001/88 requires 40 lux as a minimum illuminance, although pigs prefer dim light for resting and bright light only for defaecation [54]. The 85 lux in our study might therefore not be optimal for the pigs. Pen structuring by zones with different illuminance could improve welfare in pigs.

The tail biting outbreaks observed in this study were highly cost- and personnel intensive. Preliminary studies about straw supplementation in conventional housing systems suggested that straw provision would reduce pen mate-directed behaviour and tail biting also in pigs with intact tails [9,10,30,34,36,48,55,56]. Even small amounts of straw weighing approximately 10 to 15 g per pig per day were found to reduce tail or ear manipulation in pigs [10,48,55,57] during outbreak situations.

Less expensive enrichment material can also be effective for reducing tail biting [58]. In choice or in motivation tests, in some studies, no preferences for different (organic) materials were found [59]. In contrast to the aforementioned findings, other authors described a pig’s preference for specific material as for example corn silage [46], peat or branches [60], straw or haylage [45]. In a recent study with intact-tailed pigs in a fully slatted system, the use of multiple slat-compatible and varying enrichment (floor toy, wood post, hanging wood, fabric, hanging chew toy and different loose materials in racks or containers) could reduce the risk of tail biting efficiently [32]. These findings offer new effective combinations of enrichment, which are not restricted to straw.

It is suggested by other authors that not only one measure but multi-step intervention protocols are needed such as removal of victims and biters and provision of additional enrichment to stop the outbreak [61]. A high proportion of biters/victims in a pen was found to reduce the intervention success. With 12% biters in a pen, as observed as a maximum also in our study, the probability of intervention success was reduced to 70% [61]. This means that in our study, the preventive effect of the interactive tower alone proved insufficient to reduce tail biting to a manageable extent. The superordinate aim for the future is to define adequate preventive measures which reduce tail biting as far as possible so that the farmer’s capacity is sufficient to treat bitten pigs and to react with interventive measures.

### 4.1. Assessment of the Interactive Tower on a Conventional Farm

It can be criticised that two aspects were addressed in parallel using the tower; that of the provision of enrichment material and that of a movable object. Both aspects are combined in the concept of the “Düsser Wühlturm”, which was tested in the present study in its intended function in comparison to the stationary and empty tower serving as a simple placeholder. A 2 × 2 factorial design would have been meaningful to assess the separate impact of both functions, but due to the restrictions of conventional farm conditions (insufficient space to separate biters and victims, too few personnel to take care of a large number of injured pigs in case of a tail biting outbreak, low availability of pigs with intact tails), the tower was tested only for its intended use. The attractiveness was sustained by re-filling the tower with straw twice daily, so that the tower never ran empty, and by movability of the freely suspended plastic pipe. Five pigs could have simultaneous access to straw. In contrast to that, a straw block providing access only to two pigs simultaneously in a group of 25 pigs was found ineffective to reduce tail injuries in finishers in a previous study [30]. The specific behaviours defined for evaluation (ethogram) were selected to answer the questions whether (i) the tower remains attractive for the pigs during the entire fattening period (manipulation of tower), (ii) pen mate manipulation is decreased by the tower, and (iii) manipulation of the tower is higher than manipulation of pen mates during feeding time (behaviour outside feeding time versus during feeding). Retrospectively, an evaluation of behaviour also on days prior to severe tail biting outbreaks would have been valuable to gain information about the reason for failure of the tower as a preventive measure. To address this question, behaviour must be recorded during the entire fattening period time and the respective days could be evaluated retrospectively.

While tail biting could not be prevented, we found a reduction in tail injuries compared to pigs exposed to the stationary tower, which generally supports this strategy. Interventions taken in case of severe tail biting outbreaks (switch to interactive tower, removal of “biters”, pen division) efficiently and sustainably interrupted tail biting immediately after their implementation. Pens excluded from evaluation after interventions were observed until the end of the fattening period. Injured pigs were treated and extra straw was provided daily. By means of these measures, pigs were prevented from further injury until the end of the fattening period.

### 4.2. Behavioural Observations

In this study, it was not possible to identify individual pigs by video observation. This would have provided important information about the contribution of individual biters to the development of tail biting outbreaks on this farm. Changing the behavioural pattern at pen level might also be a valuable indicator of a forthcoming tail biting outbreak. The proportion of pigs with low tail posture in a pen was found to be an appropriate predictor [62]. Due to the fact that the entire fattening period was not recorded in our study, but only on pre-defined days, it was not possible to evaluate the days prior to a tail biting outbreak retrospectively. Automatic monitoring for reliable behavioural indicators for a forthcoming tail biting outbreak using advanced technology (Precision Livestock Farming) would allow early, preventive interventions [63,64]. The automatic detection of low tail posture as an early warning sign was successfully implemented on a commercial farm using 3D cameras [62]. To develop these tools, indicator behaviours must be defined. Observed behaviour must not be specified according to an ethogram as in our study, but can be labelled after interpretation by a veterinarian expert in ethology with for example “aggressive” or “non-aggressive” [65,66]. Algorithms for classifying social interactions were calculated successfully from the variables of body contact and orientation as well as snout contact at the head [67]. The latter was also recorded in our ethogram. Alongside chasing, head-to-head knocking, which was included in “head and neck manipulation” in our ethogram, was also found to be successful in detecting aggressive pigs [68]. Another approach was measurement of the activity index in the pen [69].

In our study, biters were detected by the farmer, and their removal led to immediate and sustained improvement. Hence, the remaining pigs did not copy the behaviour. This is in accordance with other reports stating a high variation in the rate of pigs copying biting behaviour, suggesting that an escalation in tail biting is not inevitable [33]. It has been found that biters spend more time manipulating enrichment devices [70]. In the present study, video recordings were analysed retrospectively every second week, so as to observe the onset of tail biting outbreaks and potential preliminary indicators that could have been missed. Usually, four days prior to outbreaks, activity levels start to rise [71]. Behavioural changes such as increased chewing on enrichment objects or tail biting incidents can serve as predictors prior to tail biting outbreaks six days before an outbreak occurs [42,72]. A weekly interval of behaviour evaluations might have therefore been adequate to detect early indicators of a forthcoming tail biting outbreak. Given that all measures need to be manageable and economic, investments should be made in sensitive, individual and automatic behavioural quantification systems for this purpose. Interestingly, pen mate-directed explorative behaviour without biting or fighting dominated independently of the type of tower. This could either indicate that tail biting under these conditions is not the result of aggressive behaviour or it could be assessed as a pre-injury stage of pigs directing their explorative behaviour to pen mates [7]. For final conclusions on behavioural alterations by the interactive versus stationary tower, additional studies with a larger sample size (i.e., number of pens) need to be performed.

Direct manipulation of the towers is a main parameter to evaluate its potential as an enrichment object in a barren environment. The interactive tower was used more frequently during feeding, which might be due to the increased activity level of pigs which could not eat simultaneously in this feeding system. The number of pigs per feeding place with 2.1 pigs per one feeding place was lower than the maximal number of four pigs per feeding place according to the German Animal Welfare Livestock Ordinance for this feeding system. Sensor-controlled liquid feeding systems were considered to guarantee free access to feed all the time, although periods with empty troughs occurred sporadically between feeding intervals [73]. It has to be taken into account that continuous observation of animals and feed intake curves is of high importance in this system to adapt feeding intervals immediately, if necessary (German Animal Welfare Law). Pigs are keen to manipulate the tower during feeding times due to their intrinsic motivation for synchronised feeding [74] and their intrinsic need for foraging and rooting [75,76]. Thereby, the tower may allow direct activity away from competition concerning the food by providing an alternative for animals to chew on and interact with, which could decrease stress induced by the feeding system. As a result, the type of tail biting initiated by limitations of resources may be indirectly reduced in incidence through environmental enrichment. Rooting movements triggered by the tower providing an edible substrate might support species-specific behaviour and lowered the risk of injuring pen mates. Whether moving the tower or rooting the provided straw has a higher effect cannot be ascertained. It has to be kept in mind that effective enrichment programmes specifically target the desired behaviour, which has a positive effect on the animal when presented [77].

### 4.3. Provision of Straw in Conventional Farms

Effective enrichment stimulates intrinsically motivated exploratory behaviour and triggers extrinsic reinforcement, especially if it is edible [29]. Straw meets these requirements especially well and is often provided to reduce pen mate-directed behaviour [33,34,78]. Loose straw or branches as enrichment material received the best scores from experts [26].

The availability of straw is the precondition for implementing the tower in a farm concept and has the highest impact on the economic assessment of the tower. In this study, straw with 50 mm length was used. In most husbandry systems with fully slatted flooring, long straw can interfere with the slurry system; therefore, alternatively, the effect of chopped straw was evaluated in several studies with mainly promising results. Day et al. (2008) figured out that any length of straw reduces aggression, nosing other pigs and tail biting, although long straw was more effective in reducing pen mate-directed behaviour [43]. In contrast, Lahrmann et al. (2015) did not observe significant differences in main behavioural categories such as rooting, interaction with other pigs and aggression in pigs provided with long or chopped straw [79]. The amount of straw on conventional farms with fully or partly slatted flooring should allow adequate explorative behaviour without leading to soiling of the floor and pigs. Provision of up to 80 g straw per pig per day on partly slatted floor had no negative effect on the pen hygiene [80]. A positive effect for pen structuring due to straw on the solid part of the pen as the lying area can itself support pen hygiene. The time pigs spend on straw manipulation increases with the amount of straw provided [55]. Approximately 400 g straw per pig per day is considered to fulfil the pig’s need for exploration, which was reflected in a decrease in oral manipulation of pen mates [56]. It can be assumed that straw should be loose because compressed straw blocks were not efficient to decrease agonistic behaviour [30]. In a literature review, up to 20 g straw per pig per day was appropriate to reduce tail biting by more than 80% [33].

Re-filling the tower with straw in this study could be performed quickly. The chopped straw not picked up by the pigs was compatible with the liquid manure system. Nevertheless, the return on investment of the interactive tower should be addressed in further studies. The outcome will vary depending on the time period for evaluation due to high variation in pig slaughterhouse processes and feed costs. In this study, extra costs due to installing the tower amounted to EUR 1.78 per pig. This calculation is based on an estimated service life of the tower of eight years, investment costs of EUR 380, costs for straw, an increase in working time and loss of space in the pen correlating with 0.5 fattening places. Higher average daily weight gain (~50 g) observed in the experimental compared to the control group is in accordance with results from other studies [81]. An explanation could be a higher feed intake in the experimental group or a positive influence on gastric health and hind gut microbiota. In periods of high prices for slaughter pigs this could imply for additional ~EUR 1.30 per pig (personal communication chamber of agriculture, North Rhine-Westphalia 2020). In a recent cost-effectiveness analysis, the average costs for a tail lesion in slaughter pigs were calculated depending on the prevalence of tail lesions on the respective farm. On a farm with 10% prevalence of tail injuries, costs were estimated to be EUR 2.30 and on a farm with 50% prevalence more than EUR 12. For most of the expensive measures (as straw provision), therefore, a large effect (reduction in prevalence of injuries) is required to be profitable [82].

## 5. Conclusions

The interactive tower called “Duesser Wuehlturm” encouraged exploratory behaviour in pigs by manoeuvrable object parts and the release of enrichment materials. The tower is suitable for installation on fully slatted floors. Furthermore, it is of interest for pigs during the entire fattening period and can be assessed as a tool to provide straw as a preventive measure to reduce the risk of a tail biting outbreak. Finally, in this study, tail biting could not be prevented in pigs with intact tails nor could tail injuries be reduced to a prevalence still manageable by the farmer. The study outcome supports the concept that in addition to enrichment, both detecting and removing biters by in-depth behavioural analysis of individual animals require further attention.

## Figures and Tables

**Table 1:** Summary of animals in pens allocated to groups (experimental and control) and fattening periods (batch/season) in the experiment.

Pen Number in Study Compartment	Time Period	Male	Female	Biters	Pigs with Acute Tail Injuries (Fresh Blood) *	Animal Losses	Exclusion of Pen (Fattening Week)
Experimental group (interactive straw-filled rooting tower)							
1	summer	11	14	0	3	0	
1	winter	13	12	3	18	0	
1	spring	13	12	2	16	0	week 5
2	summer	12	13	1	8	0	
2	spring	14	11	0	14	0	
3	summer	11	14	0	9	0	
3	winter	14	11	1	17	1	week 5
4	spring	16	9	2	10	0	
Control group (stationary tower without straw)							
5	summer	16	9	0	12	0	week 6
5	winter	12	13	2	24	0	week 5
5	spring	14	11	1	9	2	week 4
4	summer	11	14	1	12	0	
4	spring	13	12	3	20	0	week 2

* Acute tail injury, score 1 (Table 2).

**Table 2:** Modified German scoring system (Schwarzenauer key) used for assessing pig tails for injuries and losses [40,41].

Score	Tail Lesion	Tail Loss	Acute Tail Injury
0	Intact tail	Intact tail	No fresh blood visible
1	Superficial skin scratches (maximum width of 2 mm)	Tail loss of up to 1/3	Fresh blood visible
2	Deep injury, 2–5 mm width	Tail loss of up to 2/3	-
3	Deep injury >5 mm width	Tail loss of more than 2/3	-

**Table 3:** Ethogram used in this study modified from Zonderland et al. (2011) [42].

Behaviour ^1^	Description
Fighting	Threatening, headbutting, head-to-head pushing, knocking with the head for at least two seconds.
Manipulation of tower	Digging, sniffing, licking, chewing, manipulating tower, tower ground basis plate or straw with mouth or nose for at least two seconds.
Manipulation of head or neck of pen mate	Touching, nibbling, sniffing, licking or rooting a pen mate’s head or neck for at least two seconds.
Manipulation of trunk of pen mate	Touching, nibbling, sniffing, licking or rooting a pen mate’s trunk (belly, abdomen, back, flanks, rear end except tail) for at least two seconds.
Manipulation of tail of pen mate	Touching, nibbling, sniffing, licking or rooting a pen mate’s tail for at least two seconds.
Manipulation of limbs of pen mate	Touching, nibbling, sniffing, licking or rooting pen mate’s limb for at least two seconds.
Biting of another pen mate	Interaction of a pig using its mouth, resulting in a sudden reaction of the other bitten pig.
Biting of the head or neck of another pen mate	Interaction with another pig’s head or neck using the mouth, resulting in a sudden reaction of the other bitten pig.
Biting of the tail of another pen mate	Interaction of a pig’s tail using its mouth, resulting in a sudden reaction of the other bitten pig.

^1^ Activities which were interrupted for a time span of at least five seconds were assessed as a new action.

**Table 4:** Odds ratios and 95% Confidence Interval (CI) for the associations between the incidence of tail injuries and independent variables of the multiple logistic regression model.

Independent Variable	Odds Ratio	CI 95%	*p*-Value
Group (experimental/control)	0.31	0.17, 0.55	<0.001
Batch/Season	2.38	1.73, 3.30	<0.001
Pen	0.88	0.77, 1.01	0.075
Sex	0.99	0.58, 1.69	0.968

**Table 5:** Behavioural parameters evaluated by analysis of retrospective video recordings. Average number of specific behaviours per pig in one pen within five minutes are shown (median and range of data from all pens and all evaluated days within the respective groups).

	Observations Outside Feeding Time ^1^ and during Feeding ^2^	Experimental Group ^3^ (Interactive Tower) Median, Min–Max	Control Group ^4^ (Stationary Tower) Median, Min–Max
Fighting			
	Outside feeding time	0.005 (0–0.072)	0.004 (0–0.116)
	Feeding	0.015 (0–0.247)	0.008 (0–0.260)
Biting			
Total	Outside feeding time	0.011 (0–0.112)	0.005 (0–0.046)
	Feeding	0.016 (0–0.165)	0.012 (0–0.160)
Head and neck	Outside feeding time	0.005 (0–0.104)	0.004 (0–0.084)
	Feeding	0.009 (0–0.156)	0.008 (0–0.155)
Tail	Outside feeding time	0.004 (0–0.030)	0.003 (0–0.012)
	Feeding	0.000 (0–0.033)	0.000 (0–0.016)
Manipulation of tower			
	Outside feeding time	0.205 (0.077–0.403)	0.042 (0.013–0.683)
	Feeding	0.440 (0.132–0.694)	0.161 (0.056–0.591)
Manipulation of pen mates			
Head and neck	Outside feeding time	0.420 (0.180–0.995)	0.417 (0.220–0.683)
	Feeding	0.600 (0.267–0.976)	0.640 (0.262–1.010)
Tail	Outside feeding time	0.046 (0.016–0.218)	0.059 (0.024–0.147)
	Feeding	0.070 (0.023–0.219)	0.085 (0.037–0.151)
Proportion of time of tower manipulation to total manipulation time			
	Outside feeding time	0.244 (0.077–0.343)	0.073 (0.017–0.365)
	Feeding	0.337 (0.095–0.534)	0.142 (0.047–0.474)

^1^ Outside feeding time: As shown in Figure 5, within one hour before and one hour after feeding times at noon (12:00) and in the afternoon (17:00) three five-minute periods interrupted by 20 min were analysed (in total, 60 min per day). ^2^ Feeding: As shown in Figure 5, five subsequent five-minute periods spanning feeding times at noon (12:00) and in the evening (17:00) were analysed (in total 50 min per day). ^3^ Observation days (Wednesday in fattening weeks 2, 4, 6, 8 and 10) in pens without tail biting out-breaks (34 observations). ^4^ Observation days (Wednesday in fattening weeks 2, 4, 6, 8 and 10) in pens without tail biting out-breaks (13 observations).

## Data Availability

The datasets used and/or analysed during the current study are available from the corresponding author on reasonable request.
